# Upregulation of Hepatic Glutathione S-Transferase Alpha 1 Ameliorates Metabolic Dysfunction-Associated Steatosis by Degrading Fatty Acid Binding Protein 1

**DOI:** 10.3390/ijms25105086

**Published:** 2024-05-07

**Authors:** Jing Jiang, Hu Li, Mei Tang, Lei Lei, Hong-Ying Li, Biao Dong, Jian-Rui Li, Xue-Kai Wang, Han Sun, Jia-Yu Li, Jing-Chen Xu, Yue Gong, Jian-Dong Jiang, Zong-Gen Peng

**Affiliations:** 1 CAMS Key Laboratory of Antiviral Drug Research, Institute of Medicinal Biotechnology, Chinese Academy of Medical Sciences & Peking Union Medical College, Beijing 100050, Chinalihongying018@163.com (H.-Y.L.);; 2 Key Laboratory of Biotechnology of Antibiotics, The National Health and Family Planning Commission (NHFPC), Institute of Medicinal Biotechnology, Chinese Academy of Medical Sciences & Peking Union Medical College, Beijing 100050, China; 3 Beijing Key Laboratory of Antimicrobial Agents, Institute of Medicinal Biotechnology, Chinese Academy of Medical Sciences & Peking Union Medical College, Beijing 100050, China

**Keywords:** hepatic steatosis, glutathione S-transferase alpha 1, fatty acid binding protein 1, drug target, bicyclol

## Abstract

Metabolic dysfunction-associated steatotic liver disease (MASLD) is the most common metabolic disease of the liver, characterized by hepatic steatosis in more than 5% of hepatocytes. However, despite the recent approval of the first drug, resmetirom, for the management of metabolic dysfunction-associated steatohepatitis, decades of target exploration and hundreds of clinical trials have failed, highlighting the urgent need to find new druggable targets for the discovery of innovative drug candidates against MASLD. Here, we found that glutathione S-transferase alpha 1 (GSTA1) expression was negatively associated with lipid droplet accumulation *in vitro* and *in vivo*. Overexpression of GSTA1 significantly attenuated oleic acid-induced steatosis in hepatocytes or high-fat diet-induced steatosis in the mouse liver. The hepatoprotective and anti-inflammatory drug bicyclol also attenuated steatosis by upregulating GSTA1 expression. A detailed mechanism showed that GSTA1 directly interacts with fatty acid binding protein 1 (FABP1) and facilitates the degradation of FABP1, thereby inhibiting intracellular triglyceride synthesis by impeding the uptake and transportation of free fatty acids. Conclusion: GSTA1 may be a good target for the discovery of innovative drug candidates as GSTA1 stabilizers or enhancers against MASLD.

## 1. Introduction

Nonalcoholic fatty liver disease (NAFLD), recently replaced by the name metabolic dysfunction-associated steatotic liver disease (MASLD), is a global health problem with a prevalence of approximately 25–30% in adults [1,2]. The prevalence and incidence of MASLD have increased rapidly in parallel with the obesity epidemic, adding to the global burden of this disease [3]. The diagnostic criteria for MASLD encompass hepatic steatosis and at least one of five cardiometabolic risk factors, of which hepatic steatosis, caused by an imbalance between lipid production and removal, is a defining feature of MASLD [2,4,5]. In defining the pathogenic drivers of hepatic steatosis and metabolic dysfunction-associated steatohepatitis (MASH), a practical conceptual framework is that the capacity of the liver to process free fatty acids is overwhelmed, resulting in toxic lipid accumulation and acceleration of MASLD progression [6,7,8,9]. Currently, several potential drugs for the treatment of MASLD are in phase II or III clinical trials [10], acting on multiple targets, such as C-C motif chemokine receptor 2/5 [11], peroxisome proliferator-activated receptors α/δ [12], farnesoid X receptor [13], and apoptosis signaling kinase 1 [14]. However, due to their safety and efficacy and the complicated pathogenesis of MASLD, decades of target exploration and hundreds of clinical trials have failed, though the first drug, resmetirom, has just been approved for the management of MASH [15,16]. Therefore, there is a need for new drug candidates, especially innovative candidates with new targets.

In searching for potential new targets for the development of effective drugs against MASLD, we found that glutathione S-transferase alpha 1 (GSTA1) may be related to the accumulation of lipid droplets (LD). GSTA1, a crucial phase II metabolic enzyme for biological transformation, provides cellular protection against carcinogens and plays a role in anti-mutagenesis and anti-tumor activities [17]. However, whether GSTA1 is involved in the accumulation of LD and can be used for the development of drugs against MASLD is still unknown.

Here, we demonstrate that GSTA1 directly interacts with fatty acid binding protein 1 (FABP1) to promote the degradation of FABP1, thereby interfering with the uptake and transportation of free fatty acids, leading to inhibition of intracellular triglyceride (TG) synthesis. Overexpression of GSTA1 significantly alleviated steatosis induced by oleic acid (OA) in hepatocytes or a high-fat diet (HFD) in the mouse liver. In addition, the hepatoprotective and anti-inflammatory drug bicyclol (Bic) attenuated steatosis by upregulating GSTA1 expression. Our results highlight that GSTA1 may be a promising target for the discovery of drug candidates against MASLD.

## 2. Results

### 2.1. GSTA1 Is a Potential Regulator of Lipid Accumulation

**Figure 1:**
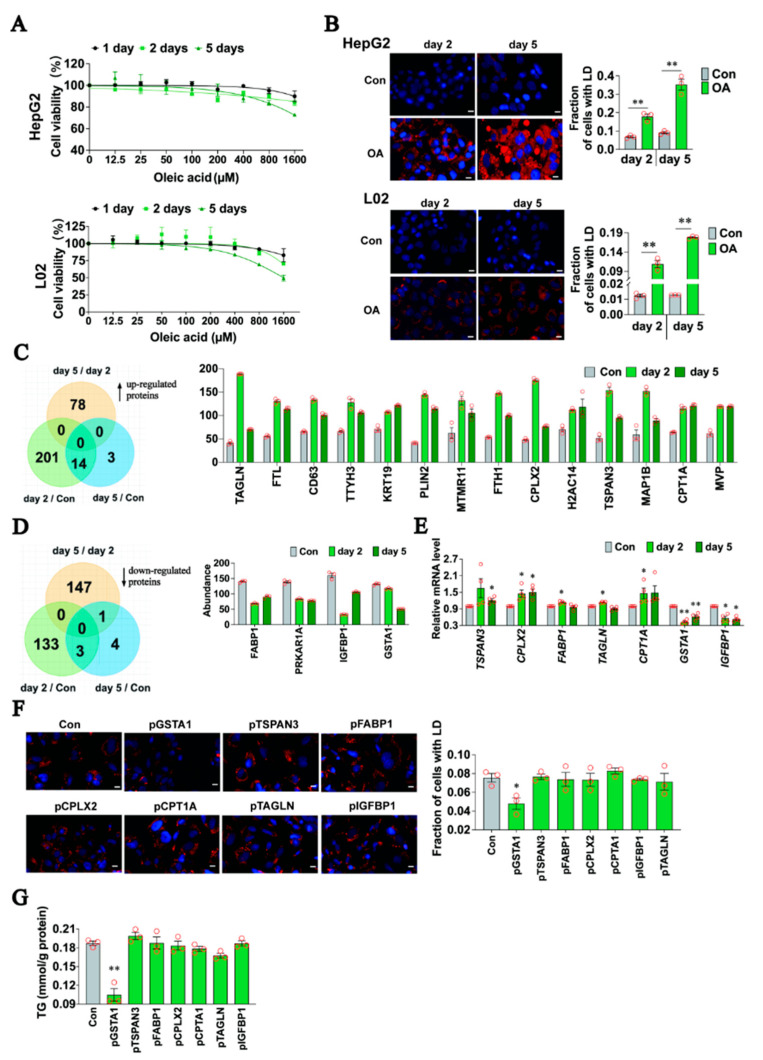
GSTA1 is a potential regulator of lipid accumulation. (**A**) Cytotoxicity to HepG2 and L02 cells detected by the CCK-8 assay. (**B**) HepG2 and L02 cells were treated with 400 µM of OA and stained with Nile red staining (red) to visualize LD. Blue, cell nucleus (DAPI). The fraction of cells with LD was quantified with Image J. Scale bar, 10 µm. (**C**–**E**) Upregulated (**C**) and downregulated (**D**) proteins were analyzed by protein sequencing, and mRNA levels were quantified by qRT-PCR (**E**) in HepG2 cells treated with 400 µM of OA. (**F**,**G**) Nile red staining (red) and quantification of LD (**F**) and intracellular TG level (**G**) in HepG2 cells transfected with plasmids followed by OA treatment. Blue, cell nucleus (DAPI). Scale bar, 10 µm. * *p*, < 0.05, ** *p*, < 0.01 *vs.* control.

To identify the potential regulator of lipid accumulation, we performed an OA-induced cellular model of hepatic steatosis in HepG2 and L02 cells. In this model, OA showed no significant cytotoxicity in 2 days but slight cytotoxicity at 400 μM in 5 days (Figure 1A). Meanwhile, the deposition of lipid droplets (Figure 1B) increased in a time-dependent manner within 5 days. Then, proteomic profiling was conducted on HepG2 cells treated with OA for 2 or 5 days to identify intracellular protein level changes associated with LD accumulation. The results showed that fourteen proteins were commonly and significantly upregulated in the cells by at least 1.5-fold compared to control cells, with no further increase with increasing treatment duration (Figure 1C). In contrast, three proteins decreased collectively and significantly by at least 0.67-fold in OA-treated cells (Figure 1D), while the abundance of another protein (GSTA1) was further reduced on the fifth day of treatment (0.89-fold and 0.39-fold, respectively, compared to the control). Seven of these proteins potentially involved in intracellular LD accumulation were quantified by qRT-PCR at the mRNA level (Figure 1E), of which gene expression of *GSTA1* was 0.43-fold at day 2 and 0.62-fold at day 5 compared to the control. To investigate the relationship between the accumulation of LD and the altered proteins, we transfected the expression plasmid into HepG2 cells. After 36 h, GSTA1, but not other proteins, led to a significant decrease in LD accumulation (Figure 1F) and intracellular TG content (Figure 1G). These results suggest that GSTA1 plays a critical role in lipid accumulation and may be a potential regulator of MASLD progression.

### 2.2. Expression of GSTA1 Is Negatively Related to the Accumulation of LD during MASLD

**Figure 2:**
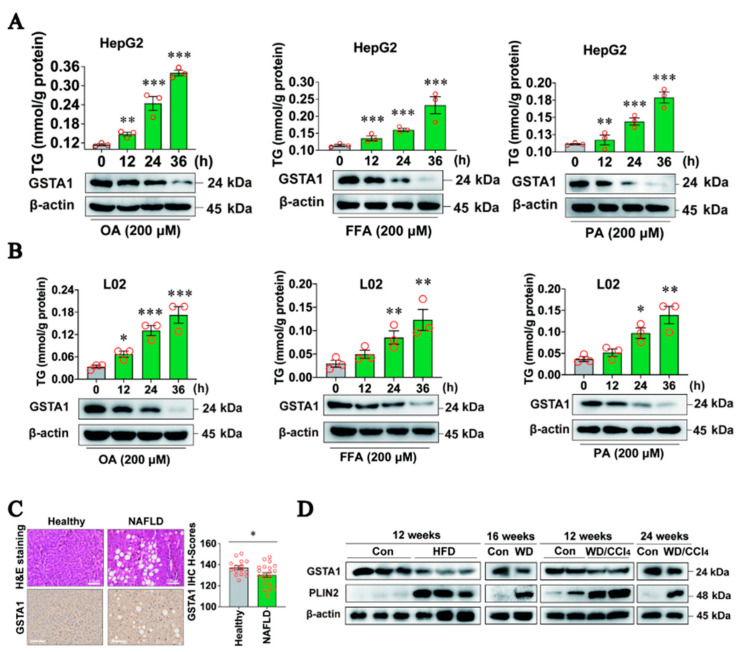
GSTA1 expression is negatively associated with the accumulation of lipid droplets (LD). (**A**,**B**) Intracellular TG and GSTA1 levels in HepG2 (**A**) and L02 (**B**) cells treated with OA, free fatty acids (FFA), or PA. (**C**) H&E staining and immunohistochemical analysis of liver samples from patients. Scale bar, 100 µm. (**D**) GSTA1 level in the liver of mice induced with HFD, WD, and WD/CCl_4_. * *p*, < 0.05, ** *p*, < 0.01, *** *p*, < 0.001 *vs.* control.

To further verify the relationship between GSTA1 and the accumulation of LD, we used hepatocytes treated with OA, free fatty acid (FFA), and palmitic acid (PA) to mimic the pathological state of MASLD in vitro. The results showed that GSTA1 levels were decreased in a time-dependent manner after treatment with OA, FFA, and PA in HepG2 cells and L02 cells (Figure 2A,B), and intracellular TG content increased accordingly, suggesting that GSTA1 is negatively related to the accumulation of LD.

Next, we further clarified the relationship between GSTA1 and the accumulation of LD *in vivo*. We detected the level of GSTA1 in liver samples from MASLD patients by immunohistochemical analysis. The results showed that the expression of GSTA1 was lower in the livers of patients with MASLD than in healthy individuals (Figure 2C). In the livers of mice fed HFD for 12 weeks, Western diet (WD) for 16 weeks, or WD/CCl_4_ for 12 and 24 weeks, the expression of GSTA1 was inversely related to the level of perilipin 2 (PLIN2) (Figure 2D), which is one of the constitutively and ubiquitously expressed proteins homing to the lipid droplets and was used as a protein marker for lipid droplets [18,19]. These results demonstrate that GSTA1 expression is negatively associated with the accumulation of LD and might be a protective factor for the progression of MASLD.

### 2.3. GSTA1 Suppresses the Accumulation of LD In Vitro

**Figure 3:**
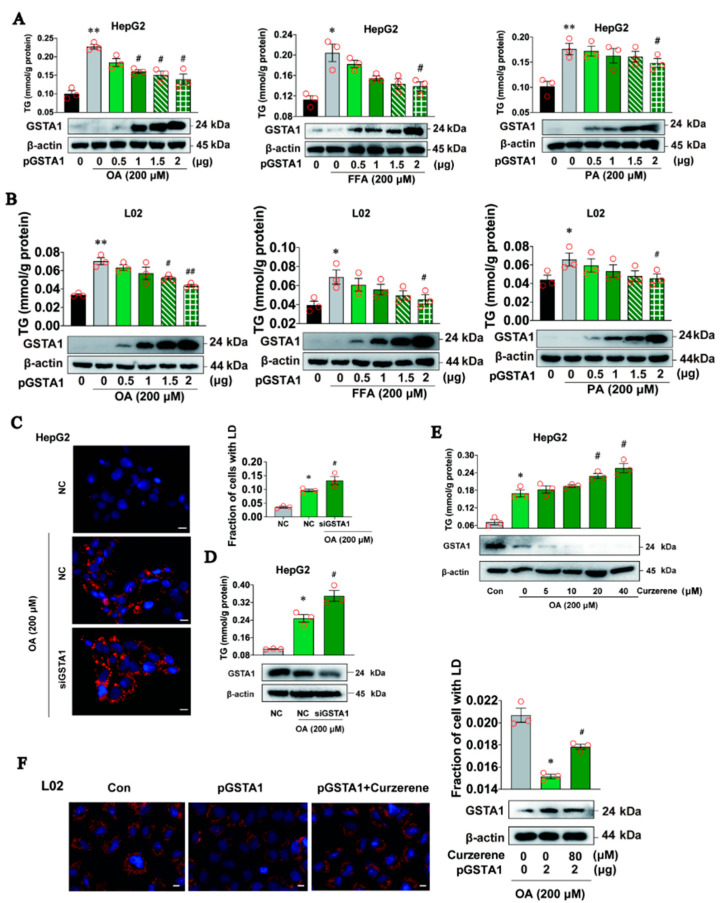
GSTA1 suppresses the accumulation of LD in vitro. (**A**,**B**) Intracellular TG and GSTA1 levels in HepG2 cells (**A**) and L02 cells (**B**) transfected with GSTA1 plasmid for 36 h and then treated with OA, PA, or FFA for 12 h. (**C**,**D**) Intracellular LD, TG, and protein levels in HepG2 cells after transfection with GSTA1-specific siRNA for 36 h and then treatment with 200 µM of OA for 12 h. The fraction of cells with LD was quantified with Image J. (**E**) Intracellular TG and GSTA1 levels in HepG2 cells treated with OA and curzerene for 24 h. (**F**) Intracellular LD and protein levels in L02 cells treated with OA and curzerene for 24 h. Scale bar, 10 µm. Blue, cell nucleus (DAPI); red, lipid (Nile red). * *p*, < 0.05, ** *p*, < 0.01 *vs*. control; ^#^ *p*, < 0.05, ^##^ *p*, < 0.01 *vs.* model control.

To further investigate the potential protective role of GSTA1 in the cellular accumulation of LD, HepG2 and L02 cells were transfected with a GSTA1-overexpression plasmid and treated with OA, FFA, or PA. The result showed that intracellular TG was significantly decreased, in parallel with the increased protein expression of GSTA1 (Figure 3A,B). In contrast, intracellular LD (Figure 3C) and TG levels (Figure 3D) were increased after disturbed expression of GSTA1 protein by GSTA1-specific siRNA (Figure 3D). Furthermore, downregulation of GSTA1 by curzerene, a specific degradation inducer for GSTA1 [20], increased the TG level in HepG2 cells (Figure 3E), while the lipid-lower role of overexpression of GSTA1 was partially reversed by curzerene in L02 cells (Figure 3F). These results collectively demonstrate that GSTA1 reduces the accumulation of LD, highlighting the potential protective role of GSTA1 in hepatic steatosis.

### 2.4. GSTA1 Reduces the Accumulation of LD in the Mouse Liver

**Figure 4:**
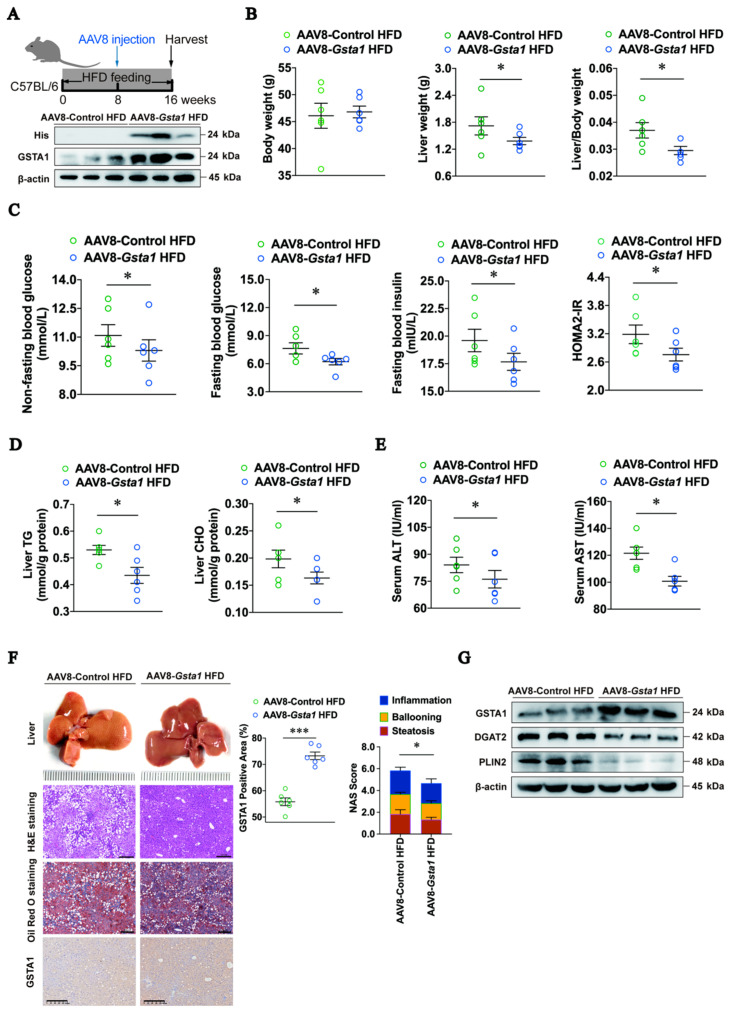
GSTA1 reduces LD accumulation *in vivo*. (**A**) Schematic of the experimental design of AAV8-Gsta1 treatment in an HFD-induced mouse model. (**B**) Body and liver weights and their ratio at week 16. (**C**) Non-fasting and fasting blood glucose concentration, fasting blood insulin concentration, and homeostasis model assessment for insulin resistance (HOMA2-IR). (**D**) Liver TG and CHO. (**E**) Serum ALT and AST. (**F**) Liver histopathology evaluated using H&E and Oil Red O staining and quantified by NAS score criteria; GSTA1 was stained by immunofluorescence staining. Scale bar, 200 µm. (**G**) Protein levels in the liver. * *p*, < 0.05, *** *p*, < 0.001 *vs.* control.

We then explored the protective role of GSTA1 for hepatic steatosis in mice induced by HFD for 16 weeks with an injection of adeno-associated virus 8 (AAV8) overexpressing GSTA1 (AAV8-*Gsta1*) at week 8 (Figure 4A). The results showed that liver weight and liver/body weight ratio were lower in mice injected with AAV8-*Gsta1*, although they had similar body weight (Figure 4B). The levels of indicators of liver metabolism, including non-fasting blood glucose, fasting blood glucose, fasting blood insulin, and insulin tolerance, were also lower in AAV8-*Gsta1*-injected mice (Figure 4C). In addition, the levels of liver TG and CHO were decreased in AAV8-*Gsta1* mice (Figure 4D). As expected, lower serum concentrations of ALT and AST were detected in mice injected with AAV8-*Gsta1* (Figure 4E), suggesting that GSTA1 also improves liver injury induced by the accumulation of LDs in these mice. Histopathological analysis further revealed that high expression of GSTA1 in mouse livers (Figure 4F, GSTA1) reduced steatosis, hepatocellular ballooning, and lobular inflammation, which was confirmed by the decreased NAS score (Figure 4F). The results were also confirmed by Western blot analysis, which showed a higher level of GSTA1 and lower levels of DGAT2 and PLIN2 (Figure 4G), two markers of LD accumulation [18,21], agreeing with our results in cells (Figure 3A,B). These results hint that a high level of GSTA1 ameliorates the accumulation of LD, thereby preventing the occurrence and progression of liver steatosis to MASLD. Therefore, upregulation of GSTA1 expression may produce a therapeutic effect on MASLD.

### 2.5. Upregulation of GSTA1 Expression by Bicyclol Attenuates Steatosis

**Figure 5:**
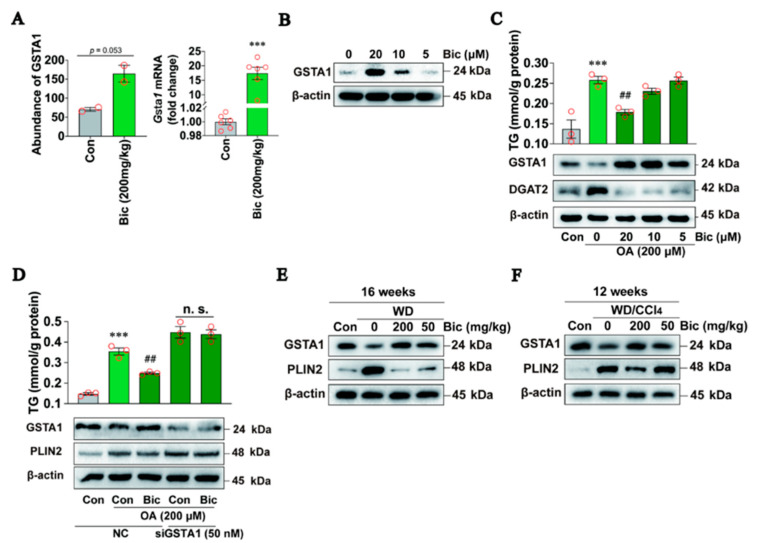
Upregulating GSTA1 expression by bicyclol attenuates steatosis. (**A**) Protein abundances quantified by protein sequencing and mRNA levels detected by qRT-PCR in the liver of mice intragastrically administered with 200 mg/kg/day of bicyclol and WD/CCl_4_ for 8 weeks. (**B**) Intracellular GSTA1 level in HepG2 cells treated with bicyclol (Bic) for 24 h. (**C**) TG and protein levels in HepG2 cells treated with OA and bicyclol (Bic) for 24 h. (**D**) TG and protein levels in HepG2 cells transfected with GSTA1-specific siRNA for 36 h and then simultaneously treated with OA and 20 µM of bicyclol (Bic) for 12 h. (**E**,**F**) Protein levels in the liver of mice treated with WD or WD/CCl_4_ for 4 weeks and then fed WD containing bicyclol for 12 weeks (**E**) or WD/CCl_4_ containing bicyclol for 8 weeks (**F**). *** *p*, < 0.001 *vs.* control; ^##^ *p*, < 0.01 *vs.* model control, n.s.: not significant.

Bicyclol, a hepatoprotective and anti-inflammatory drug widely used in the clinic to treat liver injury, has also been shown to be effective in treating hepatic steatosis in the clinic [22,23] and in animal models [24,25], but its mechanism of action remains unclear. To investigate the potential mechanism, we performed protein sequencing in the livers of mice treated orally with WD/CCl_4_ and bicyclol for 8 weeks [25] and observed by coincidence that it upregulated the expression of hepatic GSAT1 protein abundance in MASLD mice (Figure 5A, left). This result was also confirmed at the mRNA level quantified by qRT-PCR (Figure 5A, right). We speculated that the observed lipid-reducing activity of bicyclol [25,26] might be due to the induction of GSTA1 expression and thus analyzed the direct effect of bicyclol on GSTA1 expression in cells. The results showed that bicyclol increased GSTA1 expression in HepG2 cells without (Figure 5B) or with (Figure 5C) OA treatment, resulting in a dose-dependent decrease in intracellular TG levels (Figure 5C). Importantly, siRNA specifically targeting GSTA1 reduced the inhibitory effect of bicyclol on TG level (Figure 5D), indicating the critical role of GSTA1 in mediating the anti-steatosis effect of bicyclol. In mouse models of MASLD induced by WD (Figure 5E) or WD plus CCl_4_ (Figure 5F) we previously conducted [25], GSTA1 levels were reduced compared to normal control mice, whereas they were increased by bicyclol treatment, which was accompanied by decreased lipid accumulation marker protein PLIN2 (Figure 5E,F), providing further evidence that GSTA1 mediates the effect of bicyclol on liver steatosis.

### 2.6. GSTA1 Inhibits the Uptake and Transportation of Free Fatty Acids in Hepatocytes

**Figure 6:**
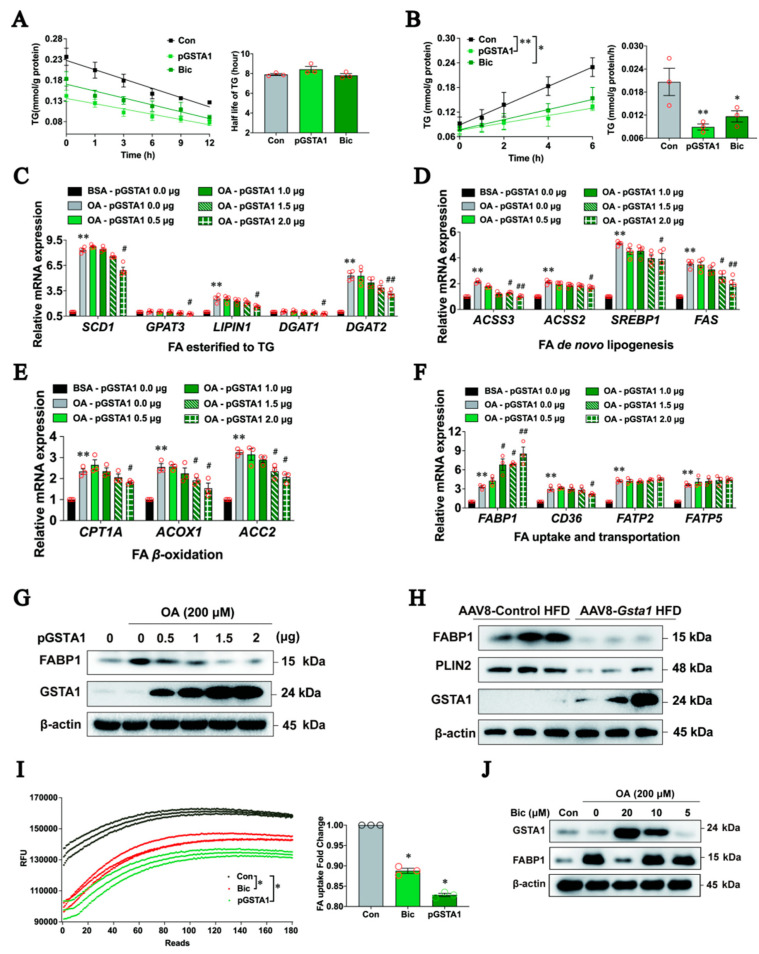
GSTA1 inhibits the uptake and transportation of FFA in hepatocytes. (**A**,**B**) Half-life of TGs in HepG2 cells (**A**) and synthesis rate in L02 cells (**B**). (**C**–**G**) mRNA levels quantified by qRT-PCR in the context of FA esterified to TG (**C**), de novo lipogenesis (**D**), beta-oxidation (**E**), and uptake and transportation (**F**), and protein levels detected by Western blot (**G**) in L02 cells transfected with the GSTA1 plasmid and treated with OA. (**H**) Protein levels in mouse livers. (**I**) FFA uptake and transportation in L02 cells transfected with the GSTA1 plasmid or treated with bicyclol (Bic). (**J**) Intracellular protein levels in L02 cells treated with bicyclol plus OA for 24 h. Experiments were performed in triplicate, and each value represents the mean ± SD. * *p*, < 0.05, ** *p*, < 0.01 *vs.* control; ^#^ *p*, < 0.05, ^##^ *p*, < 0.01 *vs.* model control.

To understand how GSTA1 influences the intracellular accumulation of LDs, we first analyzed whether GSTA1 influences the degradation or synthesis of TG. HepG2 cells were treated with OA for 12 h after 36 h of transfection with the highly expressed GSTA1 plasmids, and the intracellular TG level was analyzed after treatment with cycloheximide (CHX) [27,28]. The half-life of TGs remained unchanged after overexpression of GSTA1 or bicyclol treatment (Figure 6A), suggesting that GSTA1 has no effect on intracellular TG degradation. Next, we investigated whether GSTA1 acts on the synthesis of TGs. L02 cells were transfected with GSTA1 or control plasmids for 36 h and further treated with or without bicyclol for 12 h. Intracellular TG levels were analyzed after induction by OA containing tyloxapol, a lipoprotein lipase inhibitor that can inhibit the breakdown of triglycerides by binding to lipase [29]. Overexpression of GSTA1 or treatment with bicyclol significantly decreased the rate of TG synthesis compared to the control (Figure 6B). These results suggest that GSTA1 reduces intracellular TG synthesis but does not facilitate TG degradation.

FFA undergoes fatty acid (FA) uptake and transportation, β-oxidation, and de novo lipogenesis, and is eventually esterified to TG by enzymes residing in the endoplasmic reticulum [30,31]. We then explored which stage(s) of TG synthesis are influenced by GSTA1. In parallel with an increase in GSTA1 and a decrease in TG level after transfection with the *GSTA1* plasmid in OA-treated L02 cells, gene expression involved in the esterification of FA to TG (endoplasmic reticulum-bound stearoyl-CoA desaturase 1 (*SCD1*), glycerol-3-phosphate acyltransferase 3 (*GPAT3*), LIPIN and diacylglycerol acyltransferase 1/2 (*DGAT1*, *DGAT2*), FA de novo lipogenesis (acetyl-coenzyme A synthetase 2/3 (*ACSS2*, *ACSS3*), sterol response element-binding protein 1 (*SREBP1*) and fatty acid synthase (*FAS*)), and FA β-oxidation (carnitine palmitoyltransferase 1 alpha (*CPT1A*), acyl Co-A oxidase (*ACOX1*) and acetyl Co-A carboxylase 2 (*ACC2*)) were decreased in a dose-dependent manner (Figure 6C–E). Other genes, such as *GPAT1*, *GPAT2, APAT4*, *CPT2, ACC1*, *ACSL1*, *ACSL4*, *FATP1*, *FATP3*, *FATP4*, and *FATP6*, were not significantly altered.

On the contrary, gene expression related to FA uptake and transportation increased (Figure 6F), among which the dose-dependent upregulation of FABP1, an important factor in the FA uptake and transportation [32,33], caught our attention. However, FABP1 protein level was decreased in a dose-dependent manner in L02 cells overexpressing GSTA1 (Figure 6G). We speculated that the inconsistency between the protein and mRNA levels might be due to the feedback regulation of reduced FABP1 protein. Similarly, the FABP1 and LDs marker proteins DGAT2 and PLIN2 were decreased in the livers of HFD-fed mice co-overexpressing GSTA1 (Figure 6H), suggesting that a higher level of GSTA1 lowers the FABP1 protein and thus may influence TG synthesis by inhibiting FA uptake and transportation. Indeed, overexpression of GSTA1 in L02 cells resulted in a significant reduction in FA absorption (Figure 6I) and conversely led to decreased gene expression involved in FA esterified to TG, FA de novo lipogenesis, and FA β-oxidation (Figure 6C–E). Consistently, bicyclol decreased FA uptake and transportation in OA-treated L02 cells (Figure 6I), which was accompanied by an increased GSTA1 and decreased FABP1 protein level (Figure 6J). These results collectively indicate that downregulation of FABP1 by GSTA1 leads to inhibition of uptake and transportation of FA.

### 2.7. GSTA1 Interacts Directly with FABP1 and Promotes FABP1 Degradation

**Figure 7:**
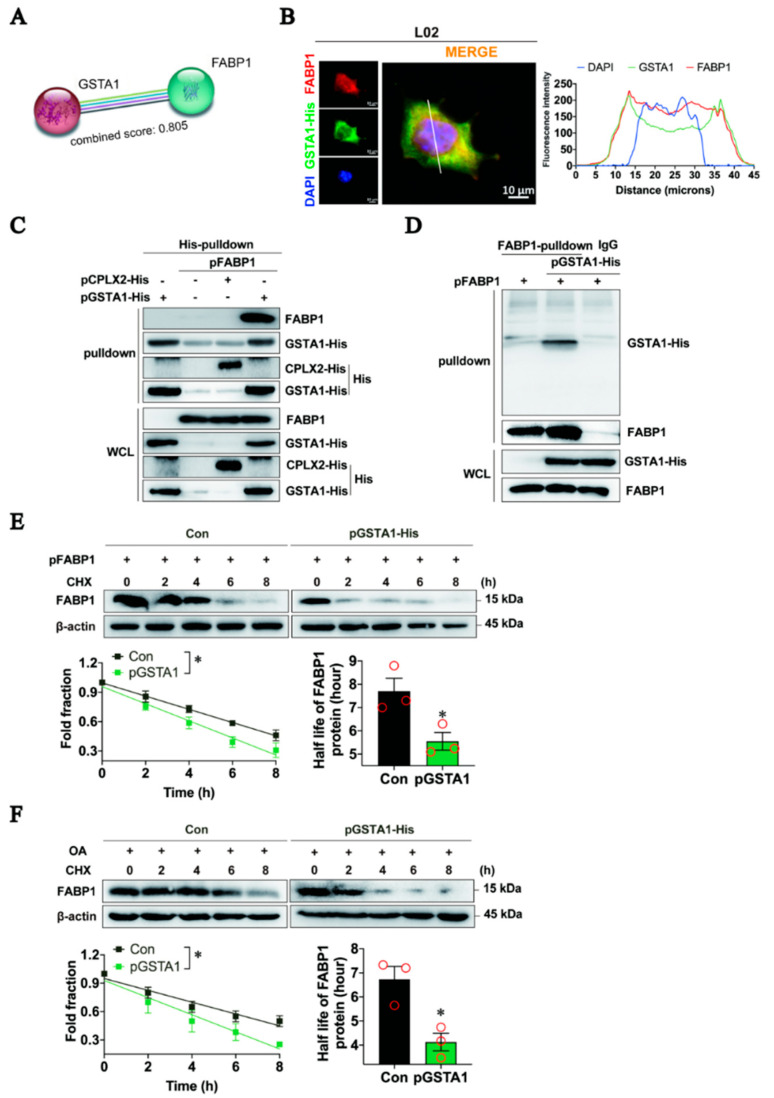
GSTA1 directly interacts with FABP1 to promote the degradation of FABP1. (**A**) Predicted GSTA1-FABP1 interaction in the database STRING. (**B**) Immunofluorescence staining of FABP1 (red), GSTA (green), and nucleus (blue) in L02 cells. Scale bar, 10 µm. (**C**,**D**) Co-immunoprecipitation analysis of protein interaction in L02 cell lysates with antibodies against GSTA1 His-tag (**C**), or FABP1 (**D**). (**E**,**F**) Half-life of FABP1 in L02 cells transfected with the FABP1 plasmid or plus pGSTA1 plasmids (**E**) or treated simultaneously with OA (**F**). Experiments were performed in triplicate, and each value represents the mean ± SD. * *p*, < 0.05; *vs.* control.

Next, we investigated the down-regulating mechanism of FABP1 by GSTA1. A clue from the website STRING [34] showed that GSTA1 can directly interact with FABP1, with a binding coefficient of 0.805 (Figure 7A). Our immunofluorescence staining results showed that GSTA1 and FABP1 were co-localized in the cytosol of L02 cells (Figure 7B). The co-immunoprecipitation results showed that FABP1 was efficiently immune-precipitated in the immune complexes with the antibody specific for His-tag (Figure 7C). Similarly, GSTA1 was readily detected in the immune complexes precipitated with the antibody against FABP1 (Figure 7D). These results suggest that there is direct binding between GSTA1 and FABP1.

We then examined the consequences of the interaction between GSTA1 and FABP1. In L02 cells overexpressed with FABP1, the total intracellular FABP1 proteins gradually declined in response to treatment with CHX, a protein synthesis inhibitor [35], whereas overexpression of GSTA1 led to an acceleration of the decline with a shortening of the half-life of FABP1 (Figure 7E). Similarly, the half-life of FABP1 in OA-treated L02 cells was also shortened by the overexpression of GSTA1 (Figure 7F). These results hint that GSTA1 directly binds to FABP1, thereby promoting the degradation of FABP1, while upregulation of GSTA1 increases mRNA (Figure 6F) but decreases the protein level of FABP1 (Figure 6G), indicating a negative feedback mechanism. However, the exact mechanism remains to be illustrated.

## 3. Discussion

**Figure 8:**
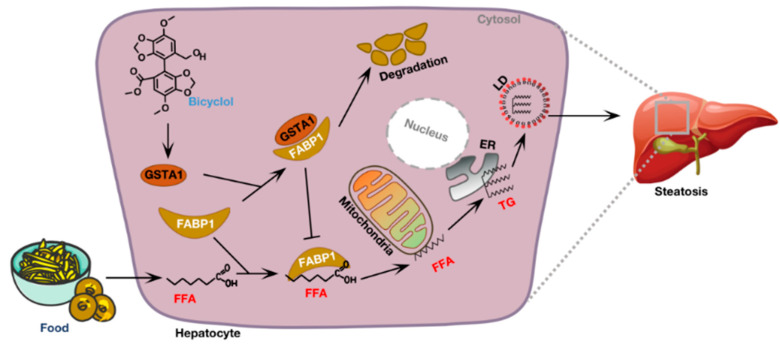
A schematic diagram depicting the mechanism of GSTA1 in hepatic steatosis. GSTA1 binds directly to FABP1, leading to the degradation of FABP1 and thus affecting the uptake and transportation of free fatty acids (FFAs) by hepatocytes. Bicyclol upregulates GSTA1, resulting in enhanced inhibition of uptake and transportation of FFAs to reduce intracellular TGs, leading to a therapeutic effect in steatosis. GSTA1, Glutathione S transferase A1; FABP1, Fatty acid binding protein1; LD, Lipid droplet; ER, Endoplasmic reticulum; TG, Triglyceride.

Currently, decades of target exploration and hundreds of clinical trials have resulted in failure, though the first drug, resmetirom has just been approved for the management of metabolic dysfunction-associated steatohepatitis [15,16,36]. Therefore, alternative pharmacological therapeutics with innovative drug targets are urgently needed. In this study, we found that GSTA1 was negatively associated with the accumulation of LD and that overexpression of GSTA1 significantly protected hepatocytes from the progression of steatosis. Mechanistically, GSTA1 directly interacted with FABP1 to promote FABP1 degradation, which impaired the uptake and transportation of free fatty acids, resulting in the inhibition of intracellular TG synthesis (Figure 8). Similarly, bicyclol stimulated the expression of GSTA1, thereby reducing hepatic steatosis, suggesting that GSTA1 may be a good target for the discovery of drug candidates against MASLD.

Glutathione S-transferases (GSTs) are a group of enzymes that catalyze the addition of glutathione to electrophilic target compounds, including carcinogens, therapeutic drugs, environmental toxins, and products of oxidative stress [37,38], thus achieving a detoxification effect by eliminating toxins and carcinogens. *In vitro* studies confirm that GSTA1 has the highest catalytic activity among human GSTs for the GSH conjugation of toxic substances and lipid peroxidation products [39]. The expression of GSTA1 was decreased in mouse models of MASLD induced by HFD-feeding [40], in mouse and patient livers [41], and in HepG2 cells treated with proinflammatory cytokines [42]. Consistent with these results, we observed decreased GSTA1 expression in the livers of MASLD mice and patients and in hepatocytes induced by free fatty acids *in vitro*. Our data suggest that hepatocyte-specific deficiency of GSTA1 exacerbates OA-induced LD accumulation, underscoring the pathogenic role of decreased GSTA1. However, whether a common or different mechanism triggers the decreased expression of GSTA1 requires further investigation, although reports have shown that this is related to the conversion of GSTA1 allele 52A to 52G, which prevents transcription factor (Sp 1) binding to the promoter site [43]. The expression of GSTA1 might be related to the level of Nrf2 [44]. Of note, GSTA1 was also previously identified as a preferential late thyroid hormone receptor beta (THR-β) responder [45]. The recently approved THR-β agonist resmetirom alleviates MAFLD through multiple mechanisms in fatty acid metabolism [16]. GSTA1 may serve as a downstream effector to exert its anti-MAFLD effects, but further clarification is needed. The plasmid for overexpression of GSTA1 and treatment with the marketed drug bicyclol, which increases GSTA1 levels *in vitro* and *in vivo*, are effective in reducing lipid droplet accumulation in our study. Therefore, in the future, developing stabilizers targeting GSTA1, creating new potential specific agonists for the activity of GSTA1 of the enzyme by elucidating the active structure of the enzyme, or performing high-throughput screening of marketed or natural products may be viable approaches for the treatment of MAFLD.

FABP1 is expressed in the liver and accounts for 7% to 10% of the total cytosolic protein [46,47]. It is mainly responsible for lipid metabolism, including fatty acid uptake, trafficking, and lipid storage [48]. Studies have previously shown that FABP1 mainly transports fatty acids to the liver and converts them into TG and phospholipids, thereby participating in intracellular fatty acid homeostasis [49,50], or directly interacts with fatty acyl-CoA synthases to donate bound long-chain fatty acids (LCFAs) for conversion to LCFA-CoAs, thereby enhancing the uptake of LCFA and cholesterol in living cells and promoting fat storage in the liver [49,51,52]. Therefore, FABP1 plays a critical role in regulating the metabolism underlying hepatic steatosis. FABP1 has been shown to be elevated in several metabolic diseases, such as MASLD, obesity, and diabetes [48,53,54], suggesting that it may be a marker or pathogenic factor for these diseases [55]. FABP1 is highly expressed in hepatocytes and is required for the uptake of FFA [56]. Increasing FABP1 expression in HepG2 cells results in increased uptake of radiolabeled oleic acid by 38% and 78%, respectively [57,58], and downregulation of FABP1 levels has a therapeutic effect in metabolic diseases [48,59]. Here, we have shown that GSTA1 binds directly to FABP1, and higher expression of GSTA1 causes degradation of FABP1, which can alleviate liver diseases. However, the exact mechanism of FABP1 degradation remains to be elucidated, as research has shown that it may be degraded by ubiquitination [48]. Also, the increase of GSTA1 by bicyclol treatment resulted in a decrease in FABP1 levels and an improvement of the disease, highlighting a possible mechanism of action of GSTA1 in metabolic diseases.

## 4. Materials and Methods

### 4.1. Cell Culture

Human hepatocyte HepG2 cells were purchased from the Cell Culture Center of the Institute of Basic Medical Sciences, Chinese Academy of Medical Sciences and Peking Union Medical College. The human hepatocyte L02 cells were kindly provided by Dr. Wei-jia Kong (Peking Union Medical College, Beijing, China). Human HepG2 and L02 cells were cultured in DMEM and RPMI1640 medium (#11995065 and #11875093, Gibco, New York, NY, USA), respectively, containing 10% fetal bovine serum (#10270-106, Gibco, New York, NY, USA) and 1% penicillin/streptomycin (P/S) (#C0222, Beyotime Biotechnology, Shanghai, China) at 37 °C and 5% CO_2_.

### 4.2. Cytotoxicity Assay

The cytotoxicity of OA in cells was assayed using the CCK-8 staining method [60]. In brief, HepG2 or L02 cells (5 × 10^3^ cells per well) were seeded in 96-well plates overnight at 37 °C and 5% CO_2_. The medium was removed, and serial twofold dilutions of OA or 10% BSA were applied for 1, 2, and 5 days. After incubation on the corresponding days, 10 µL of CCK-8 solution (#FC101-04, TransGen, Beijing, China) was added to each well and incubated at 37 °C for 2 h. The absorbance was measured at 450 nm using Enspire (Perkin Elmer, Waltham, MA, USA).

### 4.3. Nile Red Staining

HepG2 cells were plated on cell-climbing slices (#YA0350, Solarbio, Beijing, China) pre-coated with rat tail collagen in 6-well plates and then treated with 400 µM of OA for 2 and 5 days. Cells were then fixed with 3% paraformaldehyde and permeabilized with 0.5% Triton X-100 (#ST795, Beyotime Biotechnology, Shanghai, China). Nile Red (#19123, Sigma-Aldrich, Shanghai, China) solution at a concentration of 10 µM was added and incubated for 10 min. Cells were then washed with phosphate-buffered saline (PBS), sealed with a mounting medium containing DAPI staining solution (#ZLI-9557, ZSGB-BIO, Beijing, China), and dried in the dark. Images were acquired using a Zeiss Axio observer 3 microscopy (Carl Zeiss, Oberkochen, Germany) with ZEN Microscopy Software (Version 2.3, Carl Zeiss, Oberkochen, Germany) at a magnification of 63×. The fraction of cells with LD was quantified with Image J (Version 1.46, NIH, Bethesda, MD, USA).

### 4.4. Proteomic Analysis

Quantitative proteomic analysis of HepG2 cells treated with OA was performed by Shanghai Luming Biological Co. Ltd. using Tandem Mass Tags (TMT). In brief, intracellularly expressed proteins were labeled with TMT markers in vitro. Sample components were separated by liquid chromatography with high pH separation and analyzed by mass spectrometry. Mass spectrometry data were analyzed using Proteome Discover 2.4 (Thermo Scientific, New York, NY, USA). A *p*-value of <0.05 and a fold change of >1.5 or <0.67 were set as the threshold values for significantly different expressions.

### 4.5. Real-Time Quantitative RT-PCR Analysis

Total RNA in liver tissues and cells was extracted using TRIzol reagent or RaPure total RNA kit (#R4011, Magen, Guangzhou, China) according to the manufacturer’s instructions. The quality and concentrations of total RNA were determined using a NanoDrop ND-1000 spectrophotometer (NanoDrot Inc., Wilmington, DE, USA). RNA was quantified by one-step real-time quantitative reverse transcription PCR (qRT-PCR) using the HiScript II One Step qRT-PCR SYBR Green Kit (#Q221-01, Vazyme, Nanjing, China). The expression of GAPDH was used as an internal control. The primer sequences are listed in Supplementary Table S1.

### 4.6. Plasmid Construction and Transfection Assay

Full-length *GSTA1* and *CPLX2* were generated using standard PCR methods and primers (*GSTA1*-His-F: CGGGATCCATGGCAGAGAAGCCCAAGCT, *GSTA1*-His-R: CGGAATTCTTAATGATGATGATGATGATGAAACCTGAAAATCTTCC, *CPLX2*-His-F: CGGGATCCATGGACTTCGTCATGAAGCAGG; and *CPLX2*-His-R: CGGAATTCTTAATGATGATGATGATGATGCTTCTTGAACAT) and then subcloned into the corresponding pcDNA3.1(+) vectors. The plasmids *CPT1A* (#HG17390-UT), *TSPAN3* (#HG14032-CH), *FABP1* (#HG12353-UT), *TAGLN* (#HG14991-CF), and *IGFBP1* (#HG16132-CF) were purchased from Sino Biological (Beijing, China). All plasmids were confirmed by sequencing. For human protein overexpression, the plasmid was transfected into the cells with Lipofectamine 3000 (#L3000075, Invitrogen, Waltham, MA, USA) in Opti-MEM (#31985-062, New York, NY, USA). For human protein knockdown, siRNA for human *GSTA1* (#SIGS0006534-4, RiboBio, Guangzhou, China) or negative control siRNA was transfected into cells using a riboFECT CP reagent according to the manufacturer’s instructions. After a transfection time of 48 h, intracellular proteins were detected by Western blot assay.

### 4.7. GSTA1 Expression in the Liver of MASLD Mice

The liver samples we had previously obtained were used to evaluate the protein level of GSTA1 in the livers of MASLD mice or the effect of bicyclol on its expression [25,26,61]. Briefly, male C57BL/6J mice were treated with HFD (D12492, Research Diets) for 12 weeks, a Western diet for 16 weeks, or WD plus CCl_4_ injection for 12 and 24 weeks. In the bicyclol treatment experiments, bicyclol was mingled into the Western diet, equating to an oral intake of 50 mg/kg or 200 mg/kg during the respective treatment periods.

### 4.8. Construction of the Adenovirus Vector and Infection

Twelve male C57BL/6J mice (8 weeks old, 20–22 g) were obtained from SPF (Beijing) Biotechnology Co., Ltd., and randomly assigned and housed in a 12-h light/dark-light cycle with ad libitum access to water and food. To generate hepatocyte-specific *Gsta1* overexpression mice, adeno-associated virus subtype 8 (AAV8) with hepatocyte-specific expression of *Gsta1* was generated by Hanheng Biotechnology Co. Ltd. (Shanghai, China). After mice were fed with HFD (#D12492, Research Diets, New Brunswick, NJ, USA) for 8 weeks, the mice were injected once with AAV8-*Gsta1* (1 × 10^11^ genomic copies, GC/mouse) or control AAV8 into the tail vein and then fed with HFD for another 8 weeks. At the end of modeling, blood and liver samples were collected for a series of further experimental analyses.

### 4.9. Biochemical Parameter Tests

After collection of the serum and homogenization of the mouse livers, assay kits for alanine aminotransferase (ALT, #C009-2), aspartate transaminase (AST, #C010-2), TG (#A110-1), and cholesterol (CHO, #A111-1) from Nanjing Jiancheng Biotechnology Co., Ltd., Nanjing, China, were used to evaluate the corresponding biochemical parameters in 96-well plates (#701002, NEST Biotechnology, Wuxi, China) according to the instructions. Fasting and non-fasting blood glucose levels were measured using a blood glucose meter (ACCU-CHECK Performa, Roche, Switzerland) via the tail vein. Serum insulin was measured using a mouse insulin ELISA kit (#SEKM-0141, Solarbio, Beijing, China), and insulin resistance was calculated using a homeostasis model assessment-2 (HOMA2) index via an online calculator on the Diabetes Trials Unit of the University of Oxford website (https://www.dtu.ox.ac.uk/homacalculator/) (accessed on 6 April 2024).

### 4.10. Histological Examination

Paraffin-embedded liver samples were used for hematoxylin and eosin (H&E) staining to assess liver steatosis (0–3), hepatocellular ballooning (0–2), and lobular inflammation (0–3), which were performed blindly by two experts according to the NAFLD activity score (NAS) criteria [62]. Steatosis was also confirmed by Oil Red O (ORO) staining in the frozen sections. Immunohistochemical staining of GSTA1 was conducted to evaluate the expression of GSTA1 in mouse liver using the fixed liver samples and liver tissue chip of MASLD patients (#DN035Lv01 bioaitech, Xi’an, China), and then was analyzed by the H-Score indicator.

### 4.11. TG Degradation and Synthesis Assay

The assay for TG degradation was performed according to the method described previously [27,61]. In brief, HepG2 cells were transfected with GSTA1 or control plasmids and incubated for 36 h. Then, the medium was changed, and cells were induced by 200 µM of OA with or without 20 µM of bicyclol. After 12 h, the medium was replaced by normal growth medium containing 100 µg/mL of cycloheximide (CHX, #C7698, Sigma-Aldrich, Shanghai, China). Intracellular TGs were extracted at the time points indicated in the figure legends and quantified using detection kits (#E1013, Applygen, Beijing, China). Protein content in cells was quantified using the BCA protein assay kit (#23225, Thermo Scientific, New York, NY, USA). The half-life of TGs was calculated based on the fitting curve.

For the TG synthesis assay, L02 cells were transfected with GSTA1 or control plasmids for 36 h. Afterwards, the cells were treated with or without bicyclol for 12 h, followed by induction with 200 µM of OA containing 200 µM of tyloxapol (#HY-B1068, MedChemExpress, Shanghai, China). Intracellular TG and protein levels were also detected to calculate hepatic TG secretion based on the fitting curve.

### 4.12. Free Fatty Acid Uptake and Transportation Assay

Free fatty acid uptake and transportation activities were assessed using the Free Fatty Acid Uptake Assay Kit (#ab176768, Abcam) following the manufacturer’s instructions. Briefly, L02 cells were transfected with pcDNA3.1(+) or GSTA1 plasmids and incubated for 48 h. The transfected L02 cells were then seeded into a 96-well plate, allowed to adhere for 6 h, and treated with or without bicyclol for 30 min. After washing with phosphate-buffered saline, the cells were incubated in serum-free medium for 1 h and then treated with a fluorescent fatty acid mixture for 30 min. Fluorescence intensity was measured using a microplate fluorescence reader with bottom-read mode at 515 nm after excitation at 485 nm. The fluorescence signal of the control group was used as a baseline for relative quantification.

### 4.13. Histidine (His) Pull-Down Experiments

L02 cells were transfected with the indicated plasmids for 48 h. Cells were collected and lysed in Pierce IP Lysis Buffer (#87788, Thermo Scientific, Waltham, MA, USA) for 30 min at 4 °C, followed by centrifugation at 12,000× *g* for 20 min. Aliquots of proteins from L02 cells were pre-purified with Protein A/G beads (#B23202, Bimake, TX, USA) in the presence of non-specific IgG (Santa Cruz) and then incubated with His-tag antibody and 50 µL beads overnight at 4 °C with continuous mixing. The beads were washed and heated in a loading buffer at 100 °C for 5 min prior to immunoblotting assays.

### 4.14. Co-Immunoprecipitation

L02 cells were transfected with the indicated plasmids for 48 h. Cells were collected and lysed in Pierce IP Lysis Buffer for 30 min at 4 °C, followed by centrifugation at 12,000× *g* for 20 min. The supernatant was incubated overnight with 4 µg of FABP1 antibody. Magnetic beads were added to the lysate for 3 h and washed with wash buffer. Samples were boiled for 5 min and analyzed by SDS-PAGE and immunoblotting.

### 4.15. Immunofluorescence Staining

L02 cells were grown on coverslips, fixed with ice-cold methanol/acetone (1:1), and incubated for 20 min with 3% bovine serum albumin in PBS containing 0.1% Triton X-100. After washing, the cells were incubated with the primary antibody, rinsed with PBS, and immunostained with a secondary antibody. Cell nuclei were stained with DAPI. Fluorescence images were obtained by Zeiss Axio observer 3 microscopy (Carl Zeiss, Oberkochen, Germany).

### 4.16. Western Blot Analyses

Total proteins from mouse liver tissues and cells were extracted with T-PER lysis buffer (#78510, Thermo Scientific, New York, NY, USA) and M-PER lysis buffer (#78505, Thermo Scientific, New York, NY, USA) supplemented with protease and phosphatase inhibitors (#C0001 and C0004, Targetmol, Boston, MA, USA). The total protein content was quantified using a BCA protein assay kit. Protein samples were separated on gels at 12% or 15% concentrations of SDS-PAGE and transferred to PVDF membranes (#IPVH00010, Millipore, Billerica, MA, USA). The membranes were then blocked with 5% skim milk in TBST and incubated overnight at 4 °C with the respective primary antibodies (β-actin, 3700s, Cell Signaling Technology; His, #12698 and #2366, Cell Signaling Technology; GSTA1, #66624-1-Ig, Proteintech; FABP1, #13626-1-AP, Proteintech; PLIN2, #ET1704-17, HUABIO; DGAT2, #ER60651, HUABIO.). After washing with TBST, the membranes were incubated with HRP-conjugated secondary antibodies for 1 h at room temperature. Protein expression signals were visualized with ECL chemiluminescent reagent (#E1050, Beijing LABLEAD Inc.) using a ChemiDoc MP Imaging System (Bio-Rad, Hercules, CA, USA), with β-actin as an internal control.

### 4.17. Statistical Analyses

Data were presented as mean ± standard deviation (SD) and representative figures. Statistical analysis was performed using SPSS 17.0 or GraphPad Prism 8 and analyzed by the Student’s *t*-test or analysis of variance (ANOVA), followed by Student-Newman-Keuls (SNK) post hoc tests. The Kruskal-Wallis H test or Mann-Whitney U test were used for the nonparametric test. The value of statistical significance was set to *p* < 0.05.

## 5. Conclusions

In conclusion, our results indicate that maintenance of hepatic GSTA1 levels leads to a decrease in FABP1 levels, thereby improving hepatic steatosis, which represents an interesting approach for exploring GSTA1 stabilizers or enhancers as a new class of drugs against MASLD in the future.

## Data Availability

The data associated with this paper are available upon request to the corresponding author.

## Supplementary Materials

The following supporting information can be downloaded at: https://www.mdpi.com/article/10.3390/ijms25105086/s1.
