# Supplementary material for: Upregulation of Hepatic Glutathione S-Transferase Alpha 1 Ameliorates Metabolic Dysfunction-Associated Steatosis by Degrading Fatty Acid Binding Protein 1
Source: Int J Mol Sci. 2024 May 7;25(10):5086. doi: 10.3390/ijms25105086 (PMC11120891; doi:10.3390/ijms25105086)
Supplement: Supplementary file 1 [file ijms-25-05086-s001.zip › ijms-2976923-supplementary.pdf]

Supplementary Materials for

Upregulation of Hepatic Glutathione S-Transferase Alpha 1 Ameliorates Metabolic Dysfunction-Associated Steatosis by Degrading Fatty Acid Binding Protein 1

Jing Jiang [1], Hu Li [1,2,*], Mei Tang [1], Lei Lei [1], Hong-Ying Li [1], Biao Dong [1,2], Jian-Rui Li [1,3], Xue-Kai Wang [1], Han Sun [1], Jia-Yu Li [1], Jing-Chen Xu [1], Yue Gong [1], Jian-Dong Jiang [1,2,3] and Zong-Gen Peng [1,2,3,*]

[1] CAMS Key Laboratory of Antiviral Drug Research, Institute of Medicinal Biotechnology, Chinese Academy of Medical Sciences & Peking Union Medical College, Beijing 100050, China; lihongying018@163.com (H.-Y.L.)
[2] Key Laboratory of Biotechnology of Antibiotics, The National Health and Family Planning Commission (NHFPC), Institute of Medicinal Biotechnology, Chinese Academy of Medical Sciences & Peking Union Medical College, Beijing 100050, China
[3] Beijing Key Laboratory of Antimicrobial Agents, Institute of Medicinal Biotechnology, Chinese Academy of Medical Sciences & Peking Union Medical College, Beijing 100050, China
* Correspondence: lihu0112@imb.pumc.edu.cn (H.L.); pengzonggen@imb.pumc.edu.cn (Z.-G.P.); Tel.: +86-10-63166129 (Z.-G.P.); Fax: +86-10-63165281 (H.L.)

Supplementary Table S1 qRT-PCR Primer

**GENE**	**Primer（5'-3'）**	
Human *TAGLN*	Forward	AGCGTCCTGGATCTCTCTCA
	Reverse	ATGTCTGGGGAAAGCTCCTT
Human *CPT1A*	Forward	TCCAGTTGGCTTATCGTGGTG
	Reverse	TCCAGAGTCCGATTGATTTTTGC
Human *MVP*	Forward	AGGGCTTGGTGCTGTTTGATGTC
	Reverse	ATCTCCACGACCTCCACTTCCTTC
Human *FTL*	Forward	GCGATGATGTGGCTCTGGAAGG
	Reverse	TGTGGAGGTTGGTCAGGTGGTC
Human *CD63*	Forward	TTCAACGAGAAGGCGATCCATAAGG
	Reverse	CAGATGAGGAGGCTGAGGAGACC
Human *TTYH3*	Forward	TGCTGAATGGCACGGAGGTGAA
	Reverse	CTCCACGCCGTCATAGCAGAAG
Human *KRT19*	Forward	GGCGATGTGCGAGCTGATAGTG
	Reverse	GTCCCTTCCTTCCCATCCCTCTAC
Human *PLIN2*	Forward	ACCTCATGTCCTCAGCCTATCTCAG
	Reverse	CAGTCACAGTAGTCGTCACAGCATC
Human *MTMR11*	Forward	CAGCACGGTCAACGAGAGGTTC
	Reverse	CTACAGATGAATCAGGCAGGCAGAG
Human *FTH1*	Forward	TCCTACGTTTACCTGTCCATGT
	Reverse	GTTTGTGCAGTTCCAGTAGTGA
Human *CPLX2*	Forward	GATCCTGGACAATACCTCGGAG
	Reverse	CTCCACAGCATCAAGAGACTGC
Human *H2AC14*	Forward	GTGGTAAGCAGGGAGGCAAAGC

	Reverse	GCGGGATGATGCGAGTCTTCTTG
Human *TSPAN3*	Forward<br>Reverse	GCTTGGAGGCTGGATAGGCTTTG<br>CCACCTTCTTCTCAGTTCACCTTCC
Human *MAP1B*	Forward<br>Reverse	ATCTCGACACTCTGCAAGATTCT<br>TGTTTCTAAAACGTCACTTCGGT
Human *FABP1*	Forward<br>Reverse	TCGGAAATCGTGCAGAATGGGAAG<br>TGGTGATTATGTCGCCGTTGAGTTC
Human *PRKAR1A*	Forward<br>Reverse	AGCACACTGAGAAAGCGGAAGATG<br>GGCAGCACGAGGACGATTCATC
Human *IGFBP1*	Forward<br>Reverse	TTGGGACGCCATCAGTACCTA<br>TTGGCTAAACTCTCTACGACTCT
Human *SCD1*	Forward<br>Reverse	CGTTGCCACTTTCTTGCGATATGC<br>TGAAGTTGATGTGCCAGCGGTAC
Human *GPAT1*	Forward<br>Reverse	CCAGTGATGCTGCTGATGAAGGTAG<br>CGTTCCTGCTAGTGTGGGTGATTG
Human *GPAT2*	Forward<br>Reverse	TGTGGTCCAGGCGGTCATAGAG<br>AGCTTCTGGCACTGACGATGTATTC
Human *GPAT3*	Forward<br>Reverse	CGCTATTGTGTCCTACTGCCTCTG<br>TTCCCATCAAGCCGCCATGAAC
Human *GPAT4*	Forward<br>Reverse	CGTACCTGCTGCGAATGATGACC<br>GCTTCTGCTGCTCCTCCTTGAAC
Human *LIPIN*	Forward<br>Reverse	GGCTGCTAAGTCTTCTTCTCCACAC<br>TGCTCCTAAGGTCTCCAGGTCTTC
Human *DGAT1*	Forward<br>Reverse	ACTCCGAGTCTGTCACCTACTTCTG<br>GGCTATTGGCTGTCCGATGATGAG
Human *DGAT2*	Forward<br>Reverse	AGGTCATCTCAGTGCTCCAGTGG<br>CGGAAGTTGCCTGCCAGTGTAG
Human *ACSS2*	Forward<br>Reverse	TTTGCTGCTTCTGTTCTGGGTCTG<br>GTCTCTTATGGGCTGGGTTCTGTTC
Human *ACSS3*	Forward<br>Reverse	GAACAAACACTCGCCCTCTACCAG<br>ATGCCATGCTTGACCAAGACACC
Human *SREBP1*	Forward<br>Reverse	TACCACCAGCGTCTACCATAGCC<br>CTTGCGATGCCTCCAGAAGTACAC
Human *FAS*	Forward<br>Reverse	CAATTCTGCCATAAGCCCTGTCCTC<br>ACTTGGTGTTGCTGGTGAGTGTG
Human *CPT2*	Forward<br>Reverse	CGCTTTGTGCCTTCCTCTCTGTC<br>TCTCGGTTCTCACTGGTCAGGTATG
Human *ACOX1*	Forward<br>Reverse	CCGCCTGGAACTTGGAGATCATTG<br>GGTCCCGATTTCACGAATAGGTACG
Human *ACC1*	Forward<br>Reverse	TGTCCTTCTCCTCCAACCTCAACC<br>CTGCCAGCCTGTCATCCTCAATATC
Human *ACC2*	Forward<br>Reverse	GGTGAAGGAAGGTGTGGAAGTGAC<br>ATTGGTGATGAACAGGCGGATGG
Human *ACSL1*	Forward	ACTCTTCCGACCAACACGCTTATG

	Reverse	CTTCTGGATCAGTGCTGAGCCTATG
Human *ACSL4*	Forward	TGGGCTAAATGAATCTGAGGCTTCC
	Reverse	GGCGTTGGTCTACTTGGAGGAATG
Human *CD36*	Forward	GGCTGTCATTGGTGCTGTCCTG
	Reverse	TGCTGCTGTTCATCATCACTTCCTG
Human *FATP1*	Forward	ACAGTCGTCCTCCGCAAGAAATTC
	Reverse	AGCAGCTCCATTGTGTCCTCATTG
Human *FATP2*	Forward	AGCGGATTGAAGGCAGATGATGTC
	Reverse	CTCCTCGTAAGCCATTTCCCAGTG
Human *FATP3*	Forward	CAAGTTCTCGGCTGGTCAGTTCTG
	Reverse	CGCTGTCCTGTGTAGTTGATGGTG
Human *FATP4*	Forward	CAATAGCCGCATCCTGTCCTTCG
	Reverse	CTCGTCCATCACCAGCACATCAC
Human *FATP5*	Forward	AGCCCTGCCCTCTTCATCTATACC
	Reverse	TCCCACACATCAGCCCGTAGTC
Human *FATP6*	Forward	TTGGGATGACTTCTGGTTCGTGTTG
	Reverse	GGAGGGAGTTGGAGCGAATGTTG
Human *GSTA1*	Forward	CTGCCCGTATGTCCACCTG
	Reverse	AGCTCCTCGACGTAGTAGAGA
Human *GAPDH*	Forward	CGGAGTCAACGGATTTGGTCGTAT
	Reverse	AGCCTTCTCCATGGTGGTGAAGAC
Murine *Gsta1*	Forward	GCAGACCAGAGCCATTCTCAACTAC
	Reverse	AGGCTGCTGATTCTGCTCTTGAAG
Murine *Gapdh*	Forward	CTCTGGAAAGCTGTGGCGTGATG
	Reverse	ATGCCAGTGAGCTTCCCGTTCAG
